# The Treatment of Everyday Cases—Headache

**Published:** 1910-10-22

**Authors:** G. R. Bickerstaff


### THE TREATMENT OF EVERYDAY CASES—HEADACHE.

By G. R. Bickerstaff, M.R.C.S., L.R.C.P.

The great majority of cases of ordinary frontal headache in young people are due to some derangement of the digestive tract, usually constipation, and promptly yield to *continued* treatment with aperients.

The difficulty is to get people to continue taking their medicine regularly. They are so much afraid of "lowering" and weakening themselves by a constant use of laxatives, not realising how much graver is the risk of toxin-absorption, appendicitis, or other serious troubles from chronic constipation. Young girls with anæmia, domestic servants, shop-assistants, and mothers confined to housework with insufficient exercise, are all great sufferers in this way. Many of them eat very little fruit or vegetable food, and drink an insufficient amount of fluid. What they do drink of the latter is generally tea, more or less astringent from tannin.

In most of these cases, a fairly brisk purgative to begin with, such as a dose of White Mixture or a couple of hospital aperient pills, one grain of calomel in each, followed by cascara tablets, one or two twice a day with meals, will effect a great change, and the chronic headaches, tired feelings, and indigestion will probably become things of the past, *unless the cascara be neglected or forgotten*. The latter should be continued for a month or six weeks at least, the dose, one to four tablets (2-grain), being gradually decreased if possible. For some people aloin, with nux vomica and belladonna, in pills or tablets is better than cascara. Many who lead sedentary lives, or live in relaxing climates, take cascara constantly, apparently without any ill effects, and it is certainly better to continue it in this way than to run the risks from a return to chronic constipation. The tablets are more convenient for most patients than the liquid extract of cascara.

Cascara should be supplemented by drinking a sufficiency of fluid—water, hot in winter, cold in summer, at bedtime and on rising—and by eating fresh fruits, prunes and figs, green vegetables, and suet-pudding, which, when well made and light, is a valuable laxative.

For immediate relief of the headache, phenacetin and caffeine, in tablet form, is perhaps the simplest and safest remedy, a 5-grain tablet half-hourly till relieved, but not more than three or four tablets at a time. Ammonol or phenalgin are also very effective and apparently harmless, but the patient should be cautioned against taking these or any similar tablets habitually or needlessly, and contracting a habit. They will be more likely to give speedy relief if the headache is of a neuralgic character, or associated with the monthly period.

Iron should be given to anæmics, preferably combined with arsenic, and liquid cascara may be added to take the place of the cascara tablets. A good formula is:

| | |
|---|---|
| Ferri et Ammon. Cit. ... ... | gr. v. |
| Liq. Arsenicalis ... ... ... | ♏ij. |
| Ext. Cascar. Sagrad. Liq. ... ... | ♏xx. |
| Tinct. Nuc. Vom. ... ... ... | ♏v. |
| Aq. Chlorof. ad ... ... ... | ℥j. |

To mask the taste of this and other mixtures, Tinct. Chlorof. et Morph. (B.P. 1885) ♏x. will be found an excellent flavouring agent for some patients.

The aloes and iron pill of the Pharmacopœia is another good remedy for these cases—two pills, of 4 or 5 grains each, night and morning; or a smaller dose if acting too freely. So is the Mist. Ferri Sulph. of most hospital pharmacopœias. But for a long-continued treatment pills or tablets of iron will be more likely to be persevered with by the patient.

For *children's headaches*, due to stomach derangement, a small dose of salts, made effervescing by the addition of "citrate of magnesia," and taken upon waking in the morning, is very effectual, and may be followed up, daily if necessary, by sweet essence of senna (Liq. Sennæ Dulc.) or some form of liquid cascara, that combined with malt extract being as good as any.

Even better than the salts as a primary dose is calomel with powdered sugar; one grain of calomel for a child of one to four or five years old, and two grains for an older child. It is improved by the addition of two to five grains of sodium bicarbonate. Acting in addition to its aperient effect as an intestinal antiseptic, it is an invaluable occasional powder for children at the beginning of many of their troubles. Phenacetin in very small doses—2½ to 5 grains—may be tried when the headache is severe in a neurotic child over two years old as a temporary relief until the aperient has time to act. Inquiry should be made concerning injudicious diet, too many sweets or sweet cakes, or food having been "bolted," which are probable causes.

*Continued or recurrent headaches*, due to errors of refraction or organic troubles, must be remembered, but we are now speaking only of the more common forms of headache complained of in the surgery or out-patient room.

Ordinary Indigestion.

Without going into the various forms of dyspepsia and its manifold causes, it is remarkable how easily ordinary recent cases can generally be relieved, if not cured, by some such formula as this:

| | | |
|---|---|---|
| Sodii Bicarb. ... ... ... ... | | gr. x. |
| Liq. Bismuthi ... ... ... | | ʒj. |
| Tinct. Nuc. Vom. ... ... ... | | ♏v. |
| Tinct. Card. Co. ... ... ... | | ʒss. |
| Liq. Morph. Hydroch., | | |
| Acid. Hydrocyan. Dil. ... | aa | ʒss. |
| Aq. Chlorof. ad ... ... ... | | ℥j. |

taken *just after meals* three times daily.

A dose of White Mixture, or some aperient pills, should be taken at the beginning, unless there is diarrhœa. When relieved, the Morphia and Acid. Hydrocyan. Dil. may be omitted, or the medicine gradually left off, and a simple bitter stomachic substituted for it, such as:

| | |
|---|---|
| Sodii Bicarb. ... ... ... ... | gr. x. |
| Tinct. Nuc. Vom. ... ... ... | ♏v. |
| Inf. Gent. Co. ad ... ... ... | ℥j. |

This should be taken half an hour *before meals* three times daily.

In conjunction with this, aloin or cascara pills or tablets may be taken regularly if there is a tendency to constipation.

Attention to diet is, of course, necessary, the patient avoiding rich, hard, or notoriously indigestible foods; not drinking too much liquid at meals, or too much tea at any time; eating nothing between meals; and ensuring careful mastication. If the teeth are deficient a dentist should be consulted, and in the meanwhile minced or soft food taken.

