# The Treatment of Everyday Cases—Headache

**Published:** 1910-10-29

**Authors:** 


### A CORRECTION.

We regret that an unfortunate misprint occurred in Dr. Bickerstaff's article on "The Treatment of Everyday Cases—Headache" in last week's issue of The Hospital. In the prescription for indigestion, on page 108,

Liq. Morph. Hydroch.,
Acid Hydrocyan. Dil. ... aa ʒss.

should have read—

Liq. Morph. Hydroch.,
Acid Hydrocyan. Dil. ... aa ♏ijss.

The dose given originally is, of course, far in excess of that ordinarily prescribed, and the misprint will be readily detected and allowed for by medical men, but it is desirable to correct the error as soon as possible.

